# Multi-PAS domain-mediated protein oligomerization of PpsR from *Rhodobacter sphaeroides*

**DOI:** 10.1107/S1399004713033634

**Published:** 2014-02-27

**Authors:** Udo Heintz, Anton Meinhart, Andreas Winkler

**Affiliations:** a Department of Biomolecular Mechanisms, Max Planck Institute for Medical Research, Heidelberg, Germany

**Keywords:** Per–ARNT–Sim, N-cap, flanking region, AppA, coiled coil, linker, signalling helix, PAS dimer

## Abstract

Per–ARNT–Sim (PAS) domains are essential modules of many multi-domain signalling proteins that mediate protein interaction and/or sense environmental stimuli. Frequently, multiple PAS domains are present within single polypeptide chains, where their interplay is required for protein function. Although many isolated PAS domain structures have been reported over the last decades, only a few structures of multi-PAS proteins are known. Therefore, the molecular mechanism of multi-PAS domain-mediated protein oligomerization and function is poorly understood. The transcription factor PpsR from *Rhodobacter sphaeroides* is such a multi-PAS domain protein that, in addition to its three PAS domains, contains a glutamine-rich linker and a C-terminal helix–turn–helix DNA-binding motif. Here, crystal structures of two N-terminally and C-terminally truncated PpsR variants that comprise a single (PpsR_Q-PAS1_) and two (PpsR_N-Q-PAS1_) PAS domains, respectively, are presented and the multi-step strategy required for the phasing of a triple PAS domain construct (PpsR_ΔHTH_) is illustrated. While parts of the biologically relevant dimerization interface can already be observed in the two shorter constructs, the PpsR_ΔHTH_ structure reveals how three PAS domains enable the formation of multiple oligomeric states (dimer, tetramer and octamer), highlighting that not only the PAS cores but also their α-helical extensions are essential for protein oligomerization. The results demonstrate that the long helical glutamine-rich linker of PpsR results from a direct fusion of the N-cap of the PAS1 domain with the C-­terminal extension of the N-domain that plays an important role in signal transduction.

## Abstract 2

Crystal structures of two truncated variants of the transcription factor PpsR from *R. sphaeroides* are presented that enabled the phasing of a triple PAS domain construct. Together, these structures reveal the importance of α-helical PAS extensions for multi-PAS domain-mediated protein oligomerization and function.

## 1. Introduction   

Protein self-association into homodimers or higher oligomeric states is essential for the function of many proteins and confers several advantages compared with monomers (Ali & Imperiali, 2005 ▶; Matthews & Sunde, 2012 ▶). Protein oligomers are not only more resistant to protein denaturation and proteolytic degradation, but also provide additional possibilities for functional control, such as allosteric regulation or oligomer-dependent activity. The oligomerization of many signalling proteins is mediated *via* Per–ARNT–Sim (PAS) domains. PAS domains are 100–120-amino-acid structural modules that occur in all kingdoms of life. Although PAS domains can exhibit low sequence identities among each other, they share a conserved three-dimensional fold consisting of a five-stranded antiparallel β-sheet surrounded by several short α-helices (Pellequer *et al.*, 1998 ▶; Hefti *et al.*, 2004 ▶). In addition to the structurally conserved PAS core, most PAS domains possess α-helical extensions that often connect multiple PAS domains within the same protein and/or link them to a variety of effector domains such as histidine kinases, nucleotide cyclases, phosphodiesterases or DNA-binding domains (Möglich *et al.*, 2009*b* ▶). This reflects their central involvement in the regulation of a variety of biological processes, such as the maintenance of circadian rhythms, control of phototropism, nitrogen fixation and gene transcription (Taylor & Zhulin, 1999 ▶; Möglich *et al.*, 2009*b* ▶; Henry & Crosson, 2011 ▶). The α-helical PAS extensions can be found at the N-termini as well as the C-termini of PAS domains and commonly exhibit a heptad pattern of hydrophobic residues typical of amphipathic helices or coiled coils (Möglich *et al.*, 2009*b* ▶). Owing to the importance of the N-terminal helices for the stabilization of PAS dimers in a parallel arrangement and its consequences for PAS function, these elements are generally referred to as ‘N-caps’ (Ayers & Moffat, 2008 ▶). Recently, it has been proposed that helical PAS extensions that connect different domains within a PAS protein are also involved in signal transmission in addition to protein oligomerization (Little *et al.*, 2012 ▶).

Some PAS domains bind cofactors and ligands, such as haem, flavins, carboxylic acids or divalent metal ions, in a cleft formed by the inner side of the β-sheet and the short helices (Henry & Crosson, 2011 ▶). These interactions provide the possibility of sensing a plethora of different signals, which result in conformational changes of the PAS domain. These signals are then further transmitted to downstream effector domains, modulating their activity. Alternatively, PAS proteins mediate signal transduction or regulate gene transcription by protein hetero- or homo-oligomerization as, for example, in histidine kinases (Krell *et al.*, 2010 ▶), light, oxygen, voltage (LOV) proteins (Herrou & Crosson, 2011 ▶) and basic helix–loop–helix/PAS transcription regulators (Kewley *et al.*, 2004 ▶). However, although many PAS proteins such as KinA, WC-1 (White Collar 1) and ARNT contain multiple PAS domains, the molecular mechanism of multi-PAS domain-mediated protein oligomerization is only poorly understood.

**Figure 1:**
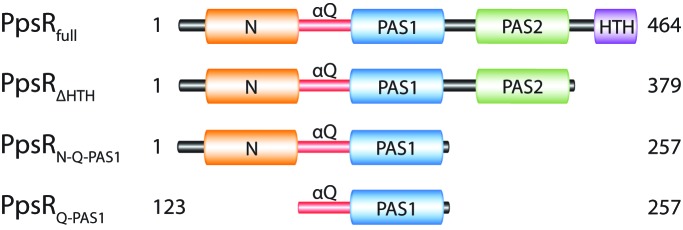
Schematic representation of PpsR_full_ and the crystallized variants. Domains are coloured as follows: N-domain, orange; Q-linker (αQ), red; PAS1, blue; PAS2, green; HTH, purple.

**Figure 2:**
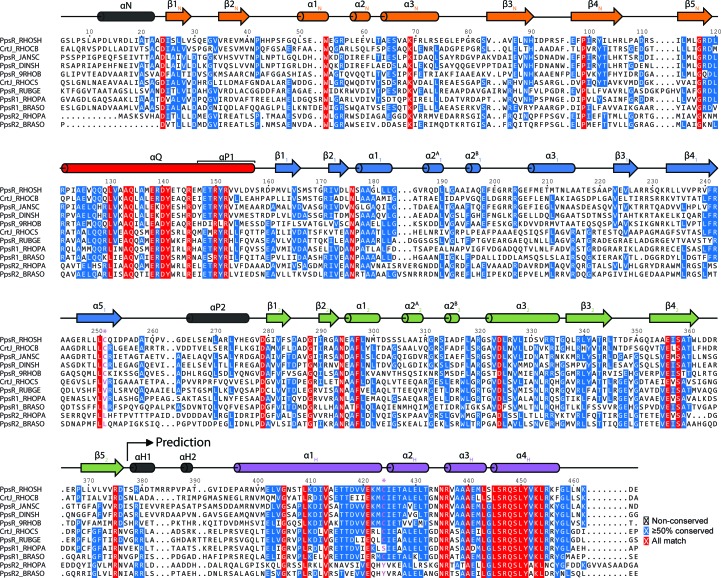
Multiple sequence alignment of PpsR proteins from different organisms. The proteins are listed according to their pairwise sequence identity to PpsR from *R. sphaeroides* in descending order. The aligned proteins are PpsR from *R. sphaeroides* (RHOSH, UniProt accession No. Q9S301), PpsR (CrtJ) from *Rhodobacter capsulatus* (RHOCB, D5ANS9), PpsR from *Jannaschia* sp. (JANSH, Q28W31), PpsR from *Dinoroseobacter shibae* (DINSH,A8LQ24), PpsR from *Thalassiobium* sp. R2A62 (9RHOB, C7DFS5), PpsR (CrtJ) from *Rhodospirillum centenum* (RHOCS, B6ITX1), PpsR from *Rubrivivax gelatinosus* (RUBGE, Q8KRL4), PpsR1 from *Rhodopseudomonas palustris* (RHOPA, Q6N9L3) and *Bradyrhizobium* sp. (BRASO, Q6A567), and PpsR2 from *Rhodopseudomonas palustris* (RHOPA, Q6N9K7) and *Bradyrhizobium* sp. (BRASO, Q8VUB5). The alignment was performed using *Jalview* (Waterhouse *et al.*, 2009 ▶) and the *MUSCLE* algorithm (default settings). The secondary-structure elements are drawn according to protomer *A* of the PpsR_ΔHTH_ structure and a *PSIPRED* (Buchan *et al.*, 2010 ▶) prediction of the secondary-structure elements in the HTH motif. The two cysteine residues Cys251 and Cys424, as well as their corresponding residues in related species, are highlighted in pink and marked with an asterisk.

The redox-responding transcription factor PpsR (photopigment suppression) from *Rhodobacter sphaeroides* (Penfold & Pemberton, 1991 ▶) is such a multi-PAS domain-containing protein (Fig. 1 ▶). PpsR possesses three PAS domains (designated N-domain, PAS1 and PAS2) and a highly conserved glutamine-rich linker (Q-linker) as well as a C-terminal helix–turn–helix (HTH) DNA-binding motif (Fig. 2 ▶). PpsR acts as a repressor of photosynthesis and tetrapyrrole-biosynthesis gene expression by binding to palindromic DNA sequences (TGTc-N_10_-gACA) present in the promoter region of PpsR-regulated genes (Moskvin *et al.*, 2005 ▶; Elsen *et al.*, 2005 ▶). It has been proposed that redox-dependent DNA binding of PpsR is regulated by the formation of an intramolecular disulfide bond (Masuda & Bauer, 2002 ▶), as well as by the binding of a haem cofactor (Yin *et al.*, 2012 ▶). Additionally, the function of PpsR is regulated in a blue-light- and oxygen-dependent manner by the antirepressor AppA (Gomelsky & Kaplan, 1998 ▶; Braatsch *et al.*, 2002 ▶; Masuda & Bauer, 2002 ▶; Han *et al.*, 2007 ▶) that, together with PpsR and its cognate DNA, is able to form a light-sensitive ternary AppA–PpsR_2_–DNA complex (Winkler *et al.*, 2013 ▶).

Here, we report the determination of the three-dimensional structure of PpsR from *R. sphaeroides*. Based on secondary-structure predictions, we designed N- and C-terminally truncated PpsR variants that comprise a single, two and three PAS domains (designated PpsR_Q-PAS1_, PpsR_N-Q-PAS1_ and PpsR_ΔHTH_ respectively; Fig. 1 ▶) to complement our crystallization approach of PpsR_full_. We solved crystal structures of the truncated PpsR variants and describe the multi-step strategy required for phasing of PpsR_ΔHTH_. The structural studies are complemented by multi-angle light-scattering (MALS) and microscale thermophoresis (MST) measurements to determine and quantify the oligomerization states of the PpsR variants in solution. In conclusion, the PpsR structures reveal an intriguing PAS-mediated protein homo-oligomerization, demonstrating that the α-helical extensions significantly contribute to protein oligomerization in addition to the PAS cores. Additionally, the observed PpsR_N-Q-PAS1_ dimer organization appears to resemble a general architecture for the connection of PAS modules with a variety of effector or sensory domains. Furthermore, the long Q-linker helix, which is the result of a fusion of the N-cap of PAS1 with the C-­terminal extension of the N-domain, features structural flexibility that plays an important role for signal transduction.

## 2. Materials and methods   

### 2.1. Cloning of full-length and truncated PpsR variants   

The gene encoding PpsR was synthesized with codon optimization for expression in *Escherichia coli* (GeneArt, Invitrogen) and was cloned into the pETM-11 expression vector using *Nco*I and *Not*I restriction sites. The resulting PpsR construct encodes the full-length protein including an N-­terminal hexahistidine tag followed by a *Tobacco etch virus* (TEV) protease cleavage site (pET-His-TEV-PpsR_full_). PpsR_Q-PAS1_, PpsR_N-Q-PAS1_ and PpsR_ΔHTH_ constructs were amplified by PCR using pET-His-TEV-PpsR_full_ as a template and the following primer pairs: 123_fw (5′-ATAT**CCATGG**GAATTGCAGAAGTTCAGCAGCAGCTG-3′) and 257_rv (5′-ATAT**GCGGCCGC** TTAATCTGCCGGATCAATCTGACACAGC-3′) for PpsR_Q-PAS1_, 1_fw (5′-ATAT**CCATGG**GAATGGGTCTGGCAGGCGGTTC-3′) and 257_rv for PpsR_N-­Q-PAS1_, and 1_fw and 379_rv (5′-ATAT**GCGGCCGC** TTAACGGCTGGTATCACGAACAACC-3′) for PpsR_ΔHTH_. The start and stop codons introduced by the forward and reverse primers, respectively, are underlined and the *Nco*I and *Not*I restriction sites are highlighted in bold. The truncated PpsR constructs were cloned into the pETM-11 vector as described for pET-His-TEV-PpsR_full_, resulting in pET-His-TEV-PpsR_Q-PAS1_, pET-His-TEV-PpsR_N-Q-PAS1_ and pET-His-TEV-PpsR_ΔHTH_, respectively.

### 2.2. Protein expression and purification   

Chemically competent *E. coli* BL21 (DE3) cells (Invitro­gen) were transformed with the different pET-His-TEV-PpsR plasmid DNAs. Single colonies grown on Luria–Bertani (LB) agar plates containing 50 µg ml^−1^ kanamycin were picked for inoculation of an overnight culture consisting of LB medium supplemented with 30 µg ml^−1^ kanamycin. The overnight culture was used to inoculate 12 l LB medium containing the same antibiotic. The cell cultures were grown to an optical density at 600 nm of 0.4 absorption units (AU) at 37°C, cooled to 18°C and the cells were further grown until the optical density reached 0.8 AU. Protein expression was induced by adding isopropyl β-d-1-thiogalactopyranoside (IPTG) to a final concentration of 0.5 m*M*. The cells were harvested by centrifugation 16 h post-induction.

All cell pellets were resuspended in buffer *A* [10 m*M* *N*-cyclohexyl-2-aminoethanesulfonic acid (CHES) pH 9.0, 50 m*M* imidazole, 300 m*M* NaCl, 5%(*w*/*v*) glycerol] including cOmplete Protease-Inhibitor Cocktail (Roche). The cells were lysed using a microfluidiser (Microfluidics) and the lysates were clarified by ultracentrifugation at 185 500*g* at 4°C for 1 h.

All hexahistidine-tagged PpsR variants were purified following the same protocol with an additional purification step for PpsR_full_. The cleared supernatant was loaded onto an Ni^2+^–NTA Superflow (Qiagen) affinity column pre-equilibrated with buffer *A*. The resin was washed with ten column volumes (CVs) of buffer *A* and the bound proteins were eluted using 5 CVs of buffer *B* [10 m*M* CHES pH 9.0, 200 m*M* imidazole, 300 m*M* NaCl, 5%(*w*/*v*) glycerol]. PpsR-containing fractions were dialysed against 1 l buffer *C* [10 m*M* CHES pH 9.0, 100 m*M* NaCl, 2 m*M* dithioerythritol (DTE), 2 m*M* ethylenediaminetetraacetic acid (EDTA), 5%(*w*/*v*) glycerol] at 4°C overnight. In parallel, the hexahistidine tag was removed from the protein using TEV protease with a 1:30 molar ratio of TEV:protein, respectively. The cleaved tag and the histidine-tagged TEV protease were removed from the PpsR-containing solutions by chromatography on Ni^2+^–NTA resin. Subsequently, the flowthrough of PpsR_full_ only was additionally loaded onto a HiTrap Heparin column (GE Healthcare) equilibrated with buffer *D* [10 m*M* CHES pH 9.0, 150 m*M* NaCl, 5%(*w*/*v*) glycerol]. After washing the column with buffer *D*, the bound protein was eluted in a gradient to 75% buffer *D* supplemented with 850 m*M* NaCl over 10 CVs. Fractions containing PpsR_full_ and the Ni^2+^–NTA flowthrough of the truncated variants were concentrated using centrifugal filter units (Amicon) and were subjected to gel filtration on a Superdex 200 column (GE Healthcare) equilibrated with buffer *D*. The protein-containing fractions were collected, concentrated, aliquoted, frozen in liquid nitrogen and stored at −80°C.

Selenomethionine-substituted PpsR_Q-PAS1_ and PpsR_ΔHTH_ proteins were expressed in *E. coli* BL21 (DE3) according to the protocol of Van Duyne *et al.* (1993 ▶), and protein purification was performed as described above.

### 2.3. Protein crystallization   

Since previous attempts to crystallize PpsR_full_ were unsuccessful, we generated C-terminally truncated PpsR variants and used them in our crystallization experiments. Initial crystallization conditions were identified in sparse-matrix screens. Subsequently, promising crystallization conditions were optimized in a hanging-drop vapour-diffusion setup using 24-well Linbro plates at 293 K. PpsR_Q-PAS1_ crystals with a hexagonal morphology appeared in 2 µl drops consisting of a 1:1 mixture of protein solution (8 mg ml^−1^) and reservoir solution consisting of 100 m*M* sodium cacodylate pH 6.5, 1.9%(*w*/*v*) polyethylene glycol (PEG) 3000, 200 m*M* MgCl_2_ after 1 d. However, individual crystals required up to 10 d to reach their maximal dimensions of ∼60 × 55 × 40 µm. Seleno­methionine-substituted PpsR_Q-PAS1_ crystals appeared in a 2 µl 1:1 mixture of protein solution (3.5 mg ml^−1^) and reservoir solution consisting of 100 m*M* 2-(*N*-morpho­lino)ethanesulfonic acid (MES) pH 6.5, 3%(*w*/*v*) PEG 4000, 200 m*M* MgCl_2_, 5 m*M* DTE and reached their final size after 2 d. For cryoprotection and also for substitution of the cacodylate buffer, crystals obtained from both experiments were incubated in a solution consisting of 100 m*M* MES pH 6.5, 10%(*w*/*v*) PEG 3000, 200 m*M* MgCl_2_, 15%(*v*/*v*) ethylene glycol for 1 min and were subsequently flash-cooled in liquid nitrogen.

PpsR_N-Q-PAS1_ crystals appeared from a 2 µl 1:1 mixture of protein solution (10 mg ml^−1^) and reservoir solution consisting of 1.6 *M* ammonium sulfate, 100 m*M* MES pH 6.5, 10 m*M* l-­cysteine, 4%(*v*/*v*) 1,4-dioxane after 1 d. Individual plate-shaped crystals reached final dimensions of ∼150 × 30 × 5 µm after 5 d but grew in clusters. However, single plates could be isolated from these clusters and were briefly incubated in reservoir solution supplemented with 20%(*v*/*v*) ethylene glycol prior to flash-cooling in liquid nitrogen.

Native as well as selenomethionine-substituted PpsR_ΔHTH_ crystals with a hexagonal morphology started to grow from a mixture of 1.1 µl protein solution (30 mg ml^−1^) and 0.9 µl reservoir solution consisting of 1 *M* ammonium sulfate, 100 m*M* Tris–HCl pH 8.5, 6%(*w*/*v*) xylitol, 12%(*w*/*v*) glycerol, 1%(*v*/*v*) dioxane after 2–3 d. Individual crystals reached their final size of ∼400 × 100 × 80 µm within two weeks. Prior to flash-cooling in liquid nitrogen, native as well as selenomethionine-substituted PpsR_ΔHTH_ crystals were incubated for 5 min in buffer consisting of 50 m*M* sodium phosphate pH 6.8, 12%(*w*/*v*) glycerol, 3.0 *M* sodium malonate pH 7.

### 2.4. Data collection, structure determination and refinement   

Diffraction data were collected from single crystals on beamline X10SA at the Swiss Light Source (Villigen, Switzerland) at 100 K. Indexing, integration and scaling of the data sets was performed using the *XDS* program suite (Kabsch, 2010 ▶). Experimental phases for the PpsR_Q-PAS1_ data were obtained from single-wavelength anomalous diffraction (SAD) experiments on crystals of selenomethionine-substituted protein using *AutoSol* in *PHENIX* (Adams *et al.*, 2010 ▶). The phases were sufficient to automatically build an initial model using *AutoBuild* in *PHENIX* (Terwilliger *et al.*, 2008 ▶) using diffraction data to 2.5 Å resolution. This model was further optimized and extended in iterative cycles of manual building using *Coot* (Emsley *et al.*, 2010 ▶) and gradient-driven maximum-likelihood refinement using *phenix.refine* (Afonine *et al.*, 2012 ▶). In the last rounds of model improvement, TLS refinement (Winn *et al.*, 2001 ▶) for two individual groups corresponding to residues 143–157 and 158–257 was included. The final model was used for phase extension to the diffraction data at 1.65 Å resolution from isomorphous native PpsR_Q-PAS1_ crystals by rigid-body refinement. This high-resolution model was again optimized by cycles of manual building and refinement similar to those described above.

PpsR_N-Q-PAS1_ was phased by molecular replacement (MR) using *Phaser* (McCoy *et al.*, 2007 ▶) in *PHENIX* with the PpsR_Q-­PAS1_ dimer as the search model. This initial dimer model consisted of the protomer in the asymmetric unit of the high-resolution PpsR_Q-PAS1_ structure and a symmetry-related molecule (symmetry operator *x* − *y*, −*y*, −*z*). The phases from MR were of sufficient quality to calculate an electron-density map based on diffraction data to 2.2 Å resolution that enabled building of the missing parts of the polypeptide chain. The structure was then further refined as described above for PpsR_Q-PAS1_ using three TLS groups for each protomer, corresponding to the N-domain (residues 6–120), the Q-linker (residues 121–157) and the PAS1 domain (residues 158–257).

The structure of PpsR_ΔHTH_ was determined using a combined MR and Se-SAD approach. Statistics of the data collection, structure refinement and model quality of PpsR_ΔHTH_ and PpsR_ΔHTH_ Se-SAD have recently been published (Winkler *et al.*, 2013 ▶). Initial phases were obtained by MR with *AutoMR* in *PHENIX* using the PpsR_N-Q-PAS1_ structure. However, a unique MR solution was only obtained when PpsR_N-Q-PAS1_ was split into two ensembles, where the first contained the N-terminal region (residues 6–141) in its dimeric form and the second encompassed a monomer of the C-terminal part (residues 145–257). Two molecules of the N-­terminal dimer and four molecules of a monomeric C-­terminal model were placed in the asymmetric unit, indicating a tetrameric PpsR_ΔHTH_ assembly. Although the electron-density map after MR was of insufficient quality for model building, the combination of MR phases (from *AutoMR*) with anomalous diffraction data (similar to that described by Schuermann & Tanner, 2003 ▶) from a selenomethionine-substituted PpsR_ΔHTH_ crystal allowed the identification of the expected 36 maxima in an anomalous difference electron-density map using *AutoSol*. After additional cycles of density modification with *RESOLVE* (Terwilliger, 2000 ▶), an interpretable high-quality electron-density map could be calculated using diffraction data to 2.8 Å resolution. A model of PpsR_ΔHTH_, extending the high-resolution structural models of the N-­domain and PAS1 domain, was built and improved in cycles of gradient-driven maximum-likelihood refinement with *phenix.refine* including secondary-structure restraints and manual building using *Coot*. Noncrystallographic symmetry (NCS) restraints were applied to the corresponding PpsR protomers *A* and *D* as well as *B* and *C* during initial rounds of model building and refinement, but were omitted during the end of the refinement to account for differences between the NCS-related protomers. TLS refinement was included [four TLS groups for each protomer, corresponding to the N-­domain (residues 6–120), the Q-linker (residues 121–157) and the PAS1 (158–255) and PAS2 domains (256–378)], during the final refinement cycles.

Statistics of the data collection, structure refinement and model quality of all structures are reported in Table 1 ▶. Model quality was analysed using the *MolProbity* (Chen *et al.*, 2010 ▶) validation tool as implemented in *PHENIX*. Atomic coordinates of the structures and structure-factor amplitudes have been deposited in the Protein Data Bank as entries 4l9e (PpsR_Q-PAS1_), 4l9f (PpsR_Q-PAS1_ SeMet) and 4l9g (PpsR_N-Q-PAS1_). The PpsR_ΔHTH_ structure was previously deposited as PDB entry 4hh2 (Winkler *et al.*, 2013 ▶). Analysis of the PpsR dimer and tetramer interfaces and the buried surface areas (BSAs) were performed using the *Protein Interfaces, Surfaces and Assemblies* service (*PISA*; v.1.42) at the European Bio­informatics Institute (http://www.ebi.ac.uk/pdbe/prot_int/pistart.html; Krissinel & Henrick, 2007 ▶). Illustrations of the obtained structures were prepared with *PyMOL* v.1.3r1 (Schrödinger).

### 2.5. Multi-angle light-scattering experiments   

Individual PpsR variants were analysed using an HPLC (Waters) setup including a Superdex 200 10/300 GL column (GE Healthcare) connected to a MALS detector (DAWN HELEOS, Wyatt Technology) combined with a refractive-index detector (Waters). The system was equilibrated with buffer *D*, and 400 µl of a 20 µ*M* protein solution containing the respective PpsR variants was loaded and fractionated at a flow rate of 0.5 ml min^−1^. Data analysis was performed using the *ASTRA* software provided by the manufacturer (Wyatt Technology).

### 2.6. Microscale thermophoresis   

The oligomerization of PpsR_full_ and the truncated variants was quantified by microscale thermophoresis (MST) using a Monolith NT.115 (Nanotemper). The proteins were randomly labelled at their amine positions using NT-647 red fluorescent dye (Nanotemper), with approximately one label per monomer, as described in the Monolith NT protein labelling kit RED-NHS provided by the manufacturer (Nanotemper). 1:2 dilution series of unlabelled proteins were prepared over an appropriate concentration range using buffer *D* and were mixed with equivalent volumes of labelled proteins, resulting in final concentrations of 12.5 n*M* for PpsR_full_ and PpsR_ΔHTH_ as well as 10 n*M* for PpsR_N-Q-PAS1_ with respect to the labelled species. Standard treated capillaries (Nanotemper) were used for all experiments. Data for three individual experiments were averaged and evaluated using the quadratic equation of the law of mass action with the constraint of a fixed labelled species concentration.

## 3. Results   

### 3.1. Structure determination of PpsR_ΔHTH_   

**Figure 3:**
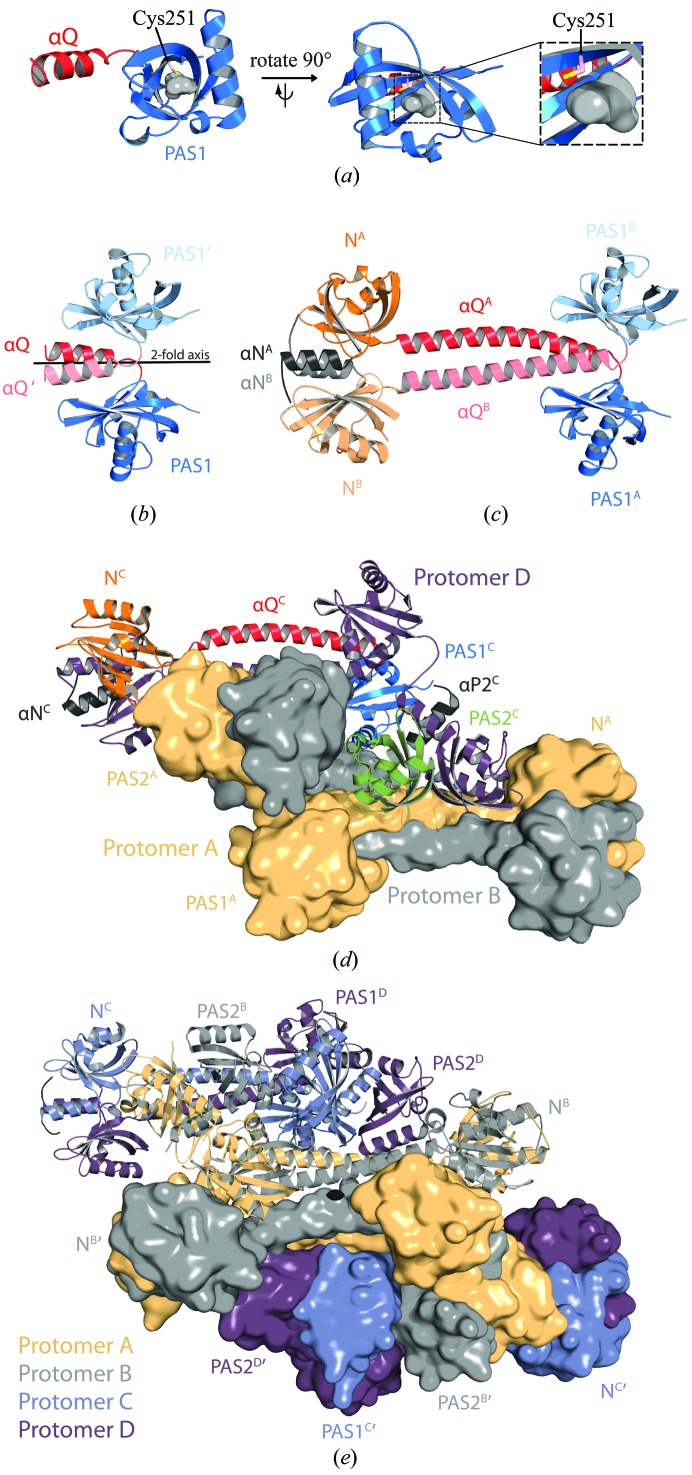
(*a*) Cartoon representation of PpsR_Q-PAS1_. The PAS1 domain is coloured blue and the structured C-terminal part of the Q-linker helix is coloured red. Cys251 (pink) located in strand β5_1_ (Fig. 2 ▶) points towards a small cavity (shaped as a grey surface) that is frequently involved in cofactor binding of PAS domains. (*b*) Structure of a parallel PpsR_Q-PAS1_ dimer. The PpsR_Q-PAS1_ protomer is coloured according to (*a*), whereas the symmetry-related molecule (distinguished by a prime) is coloured in equivalent but lighter colours. The crystallographic twofold axis generating the dimer is shown in black. (*c*) Structure of the PpsR_N-Q-PAS1_ dimer. The N-domain of PpsR_N-Q-PAS1_ is coloured orange and its capping helix grey. The remaining structural elements are coloured according to (*a*). Protomer *A* is coloured in saturated colours and protomer *B* in light colours. Individual domains of different protomers are distinguished by superscript letters. (*d*) Structure of the PpsR_ΔHTH_ tetramer (PDB entry 4hh2; Winkler *et al.*, 2013 ▶) formed by two antiparallel dimers. Protomer *C* is coloured according to (*c*) and the additional PAS2 domain is shown in green. The dimer formed by protomers *C *and *D* (coloured purple) is shown as a cartoon representation, whereas that formed by protomers *A* (coloured light orange) and *B* (coloured grey) is depicted as a surface representation. (*e*) The octameric assembly of PpsR_ΔHTH_ represented by two tetramers generated by a crystallographic twofold axis (Winkler *et al.*, 2013 ▶). View along the twofold symmetry axis perpendicular to the plane (black symbol). One tetramer is shown as a cartoon representation and the second as a surface representation. Protomers *A* are coloured in light orange, *B* in grey, *C* in slate and *D* in dark purple, respectively. Individual domains of different protomers are distinguished by superscript letters and labels of the symmetry mate are marked with an additional prime. Note that the N-Q-PAS1 domains of protomers *A* and *B* exclusively stabilize the quaternary assembly.

All initial attempts to solve the phase problem of PpsR_ΔHTH_ (see Fig. 1 ▶ for domain architecture) by MR using models of homologous PAS domains failed, likely owing to the low sequence conservation (identities of below 25% with respect to the PAS domains of PpsR). Furthermore, *ab initio* phasing of diffraction data collected from selenomethionine-substituted PpsR_ΔHTH_ crystals using *HySS* in *PHENIX* (*AutoSol*; Grosse-Kunstleve & Adams, 2003 ▶), *autoSHARP* (Vonrhein *et al.*, 2007 ▶) and *SHELX* (Sheldrick, 2008 ▶) was unsuccessful owing to the weak anomalous signal below 6 Å (Winkler *et al.*, 2013 ▶). We therefore applied a multistep divide-and-conquer strategy to successfully determine the structure of the triple PAS protein PpsR_ΔHTH_. We crystallized PpsR variants that encompass one or two PAS domains, determined their structures and used these models for phasing the diffraction data of longer variants by MR. In a first step, we determined the structure of PpsR_Q-PAS1_ comprising the glutamine-rich linker (Q-linker) and the PAS1 domain (amino acids 123–257; Fig. 1 ▶). Phasing was performed by Se-SAD and an initial model was built (Table 1 ▶). We finally extended the phases to 1.65 Å resolution using a native data set and refined a high-resolution structure for this variant (Table 1 ▶). This model comprised residues 143–257 of one polypeptide chain (Fig. 3 ▶ *a*) in the asymmetric unit. The 20 N-­terminal residues are disordered and could not be modelled into the electron-density map. The remaining ordered part of the Q-linker region (residues 143–157) forms a helical element that corresponds to a classical PAS N-cap. Together with a molecule related by crystallographic dyad symmetry, PpsR_Q-PAS1_ forms a PAS dimer which features an assembly common to N-cap-comprising PAS domains (Ayers & Moffat, 2008 ▶; Fig. 3 ▶ *b*).

In a second step, we determined the structure of a PpsR variant comprising the N-terminal domain, the Q-linker and the PAS1 domain (PpsR_N-Q-PAS1_, amino acids 1–257; Fig. 1 ▶) at 2.2 Å resolution (Table 1 ▶). Phases for PpsR_N-Q-PAS1_ were obtained by MR using the PpsR_Q-PAS1_ dimer as a search model. Since the dimer arrangement of the PAS1 domain was virtually identical in both crystal forms, we obtained excellent phases and could model the remaining parts of the Q-linker and the N-domain into the electron-density map. The final model of PpsR_N-Q-PAS1_ contains a dimer in the asymmetric unit comprising residues 6–246 for protomer *A* and 8–250 for protomer *B* (Fig. 3 ▶ *c*). Poor electron density was only observed in the β3_N_–β4_N_ loop (secondary-structure elements of individual PAS domains are numbered β1–β5 and α1–α3, with subscripts indicating the corresponding domain: N, N-domain; 1, PAS1 domain; 2, PAS2 domain; Fig. 2 ▶) in the N-domain of protomer *B*.

The structure of the PpsR_ΔHTH_ construct, which encompasses the N-domain and the PAS1 and PAS2 domains, was finally determined to 2.8 Å resolution using a combination of MR and experimental phases. Individual domains of the PpsR_N-Q-PAS1_ dimer were used as search models for MR and the obtained initial phase information was subsequently used for the identification of Se positions in the PpsR_ΔHTH_ Se-SAD data (Winkler *et al.*, 2013 ▶). Crystallographic analysis of the triple PAS protein PpsR_ΔHTH_ revealed four polypeptide chains in the asymmetric unit forming an intricate tetrameric assembly (Fig. 3 ▶ *d*). Whereas unambiguous and interpretable electron density was observed for most regions of promoters *A*, *B* and *C*, weak electron density was observed for parts of the N-domain and PAS1 domain of protomer *D*. These parts were modelled taking into account information from the higher resolution structures and protomer *A* of the PpsR_ΔHTH_ structure that is related to protomer *D* by noncrystallographic symmetry. Ambiguous electron density was also observed in the loop connecting the PAS1 and PAS2 domains around residues 258–262 for protomers *A*, *B* and *C*, suggesting high flexibility of this region. However, this region could be modelled in protomer *D*, and superposition with the other protomers enabled the correct assignment of all PAS2 domains to the individual polypeptide chains. Finally, the five N-terminal residues as well as the C-terminal arginine of all protomers were disordered and could not be modelled into the electron-density map.

### 3.2. Structural comparison of PpsR_Q-PAS1_, PpsR_N-Q-PAS1_ and PpsR_ΔHTH_   

The PpsR_Q-PAS1_ structure revealed a typical PAS fold consisting of a five-stranded antiparallel β-sheet flanked by several helices. In addition to the PAS1 core, part of the Q-­linker adopts an α-helical conformation corresponding to a PAS N-cap. This is important for dimer formation as observed for other N-cap-containing PAS domains (Ayers & Moffat, 2008 ▶). In addition to the evolutionary conservation of such a PAS dimer, analysis of the contact area using the *PISA* web server (Krissinel & Henrick, 2007 ▶) revealed a buried surface area (BSA) of 2590 Å^2^ (Fig. 3 ▶ *b*) and supported the crystallo­graphic dimer as biologically relevant. The main dimerization interface is formed by the N-cap, its connecting loop to the PAS core (residues 156–161) and β5_1_ (Supplementary Fig. S1^1^). The N-cap further contributes to PpsR_Q-PAS1_ dimer formation by protecting hydrophobic patches on the PAS core of the symmetry-related molecule (Supplementary Fig. S2), as also reported for a subset of other PAS proteins (Ma *et al.*, 2008 ▶). This structure additionally revealed that Cys251, which has been reported to form an intramolecular disulfide bond with Cys424 (Masuda & Bauer, 2002 ▶), is located in the middle of β5_1_, pointing towards a small cavity inside the PAS1 domain (Fig. 3 ▶ *a*).

In line with the parallel PpsR_Q-PAS1_ dimer, we observed a dimeric arrangement of the two protomers in the PpsR_N-Q-PAS1_ structure (Fig. 3 ▶ *c*). The PpsR_N-Q-PAS1_ dimer has a barbell-like quaternary structure with a protomer crossing along a central imperfect dyad axis. Owing to the protomer crossing, the N-­domain and PAS1 domain of a single protomer are located on opposite sides of the dyad axis. The functional relevance of the observed dimer architecture is supported by a large number of contacts, which lead to a total BSA of 6180 Å^2^. The PpsR_N-Q-PAS1_ structure also allowed the identification of an additional PAS domain in the N-terminal part of PpsR, which was predicted based on sequence information from some PpsR homologues (Kovács *et al.*, 2005 ▶). The PAS core is N-­terminally flanked by a helix (αN), which again corresponds to a classical PAS N-cap and constitutes a central part of the N-domain dimer interface (Supplementary Fig. S2), in analogy to the N-cap of PAS1. The highly conserved Q-linkers (Fig. 2 ▶) form a ∼57 Å long coiled-coil-like structure which connects the N-domain dimers to the PAS1 dimers.

While the arrangement of the structural elements in the PpsR_N-Q-PAS1_ structure is essentially the same as in the PpsR_ΔHTH_ structure, the latter revealed the importance of the PAS2 domain of PpsR. As observed for the N-domains and the PAS1 domains, the PAS2 domains also form a homodimer and contribute significantly to the overall PpsR dimer interface by the interaction of highly conserved residues located in the PAS2 β-sheets (Glu356 with Arg375) as well as in β4_2_ and the loop connecting the N-terminal capping helix of PAS2 (αP2) and β1_2_ (Asp279 with Ser358; Supplementary Fig. S1). In addition, the PAS2 N-cap also contributes to dimer formation by interacting with the corresponding N-cap of the second protomer. Notably, His275, which has been proposed to be involved in the coordination of a haem cofactor (Yin *et al.*, 2012 ▶), is located on the PAS2 N-cap. However, the PAS2 domains are not only involved in PpsR dimer formation; they also mediate the formation of an intertwined PpsR tetramer formed by two PpsR dimers arranged head to tail (Fig. 3*d* ▶). The PAS2 domains of each dimer interact with the Q-linkers of the other dimer, thereby mediating PpsR tetramer formation. Highly conserved residues of PpsR are involved in this interaction, including elements of helix α3_2_ as well as β3_2_ of PAS2 and the Q-linker (such as, for example, Arg339 with Glu138 and Leu340 with Gln134; see Fig. 2 ▶). Since the PAS2 dimer axis does not align with that of the N-­domain and PAS1 dimers, noncrystallographic symmetry is only observed for promoters *A* and *D* as well as for promoters *B* and *C*. Analysis of the contact surfaces using *PISA* supported the relevance of the observed PpsR_ΔHTH_ tetramer, with a BSA of 26 870 Å^2^. However, in addition to the tetramer interface, the Q-linker provides another antiparallel oligomerization interface with a symmetry-related molecule (symmetry operator −*x*, −*x* + *y*, −*z* + 2/3). This interaction results in the formation of a PpsR octamer (Fig. 3 ▶ *e*) mediated by contacts of the N-domains, the Q-linkers and the PAS1 domains of the crystallographic and symmetry-related protomers *A* and *B*. The BSA of the octamer interface is 27 160 Å^2^, which is comparable to that of the tetramer interface (BSA of 26 870 Å^2^). Additional stabilization by the octamer interface is likely to be one reason for the better defined electron density observed for several regions of protomer *A* in comparison to protomer *D*.

**Figure 4:**
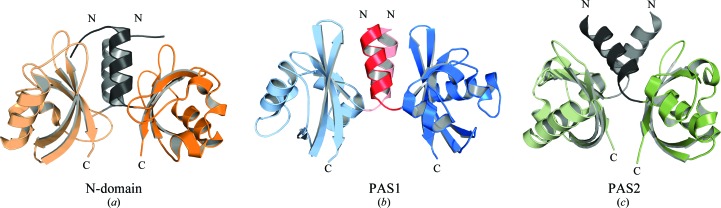
Structures of the three different PpsR PAS domain dimers including their N-caps (from PpsR_ΔHTH_ protomers *A* and *B*). The PAS core and N-cap of one protomer is coloured according protomer *C* in Fig. 3 ▶(*d*), whereas the second protomer is coloured in equivalent but lighter colours. (*a*) N-domain dimer (residues 6–120). (*b*) PAS1 dimer (residues 147–256). (*c*) PAS2 dimer (residues 264–378). The N- and C-termini of each domain are labelled.

**Figure 5:**
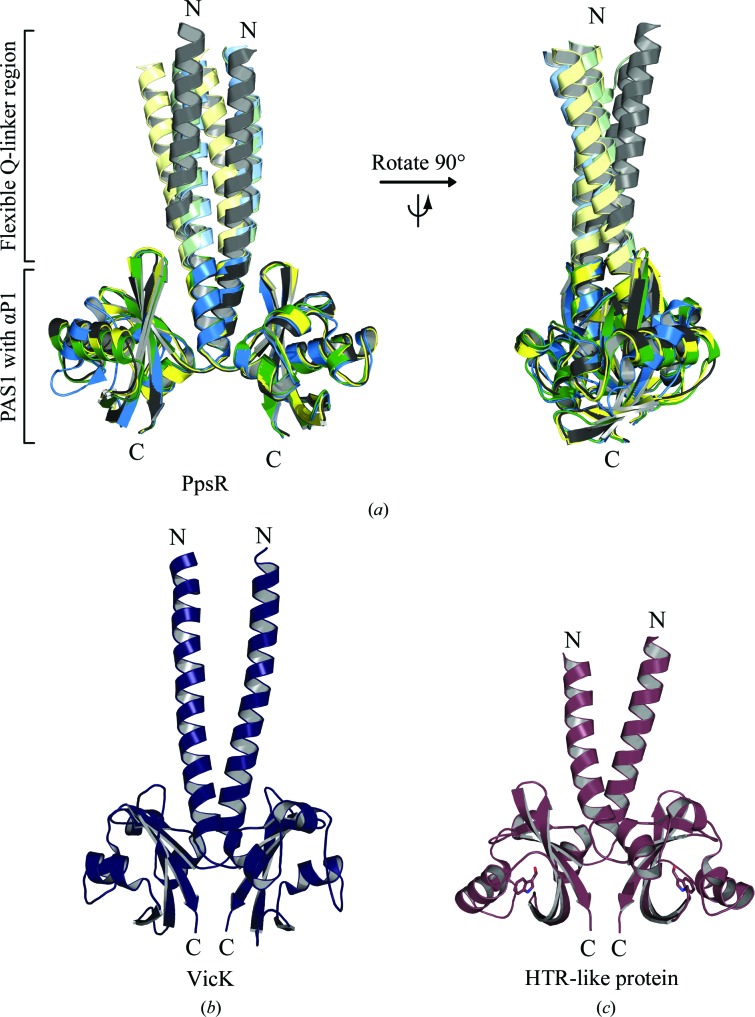
(*a*) Structural superposition of the PpsR Q-PAS1 dimers of PpsR_ΔHTH_ (protomers *A* and *B*, blue; protomers *C* and *D*, green), PpsR_N-Q-PAS1_ (yellow) as well as the recently published AppA–(PpsR)_2_ complex structure (black; PDB entry 4hh3; Winkler *et al.*, 2013 ▶). The PAS1 dimers and their corresponding N-caps are rigid dimer modules (bright colours) that exhibit an identical conformation in all structures, whereas the corresponding N-terminal helical regions of the Q-­linkers (pale colours) show different relative orientations. Even in the AppA–(PpsR)_2_ complex structure, where the asymmetric binding of AppA to PpsR induces significant structural changes in the conformation of the Q-linker (Winkler *et al.*, 2013 ▶), the conformations of the PAS1 and N-­domain dimers, including their N-caps, are unchanged. (*b*) The structure of the S-helix PAS domain dimer of VicK (PDB entry 4i5s; Wang *et al.*, 2013 ▶) shows a very similar conformation to that observed for the Q-linkers and PAS1 domains of PpsR. For reasons of clarity, the N-terminal HAMP and the C-terminal S-­helix and DHp domains are not shown. (*c*) The structure of the S-helix PAS domain dimer of HTR-like protein (PDB entry 3bwl; protomers *A* and *B*) also exhibits a comparable conformation to that observed for PpsR and VicK. The PAS domains of the HTR-like protein have an 1*H*-indole-3-carbaldehyde ligand that is shown as stick model.

Superpositions of identical PAS domains within the protomers of all three PpsR crystal structures showed a very similar three-dimensional conformation of the N-domain and the PAS1 and PAS2 domains. Pronounced differences were only observed for helix α3_1_ of the PAS1 domain of protomer *A* (PpsR_ΔHTH_), which is rotated by about 42° compared with all other PAS1 domains (Supplementary Fig. S3). This rotation is induced by contacts with a symmetry-related molecule in the octamer interface of the PpsR_ΔHTH_ structure. Comparisons of the N-domain, PAS1 and PAS2 domain dimers, including their N-caps, identified these structures as rigid dimer modules (Fig. 4 ▶). Although molecular details of the dimerization interface differ between the various PAS dimers, their dimer architecture is comparable. The N-caps constitute a central part of all dimer interfaces by protecting hydrophobic patches on one side of the PAS β-sheet. However, the interactions between the PAS2 N-caps are not as extensive as those observed for the N-caps of the N-domain and PAS1 domain. In contrast to the N-domain and PAS1 domains, the PAS2 domains make additional dimer contacts *via* their β-sheets (*e.g.* Glu356 with Arg375; Supplementary Fig. S1). Superpositions of all determined PpsR structures based on their PAS1 dimers revealed diverse orientations of the N-terminal helical part of the Q-linker, which results in different relative arrangements of the N-domain and PAS1 domain dimers in the determined PpsR structures (Fig. 5 ▶ *a*). This is the result of inherent flexibility in a distinct hinge-like Q-linker region (residues 142–146), which is further reflected in subtle differences between the Q-linkers of the symmetrically arranged PpsR_ΔHTH_ protomers *A* and *D* as well as *B* and *C* (Fig. 5 ▶ *a*). In contrast, the PAS1 cores and their corresponding N-caps are rigid modules that exhibit an identical conformation in all determined PpsR structures. Since α-­helical extensions are not only present at the N-termini but also at the C-termini of some PAS domains (Möglich *et al.*, 2009*b* ▶), this suggests that the long Q-linker helix results from a direct fusion of a C-terminal α-helical extension of the N-­domain with the rigid N-cap of the PAS1 domain. Protein-sequence analysis suggests that such long helices, also termed signalling helices (S-helices; Anantharaman *et al.*, 2006 ▶), frequently occur in multi-domain signalling proteins and form coiled coils, as observed for the Q-­linker of PpsR. These S-­helices usually connect an N-­terminal sensory domain with a C-terminal effector, or two sensory domains (Anantharaman *et al.*, 2006 ▶). Similarly to PpsR, the sensor histidine kinase VicK from *Streptococcus mutans* also possesses a long S-helix, which connects a HAMP and a PAS domain (Wang *et al.*, 2013 ▶). The structures of the PpsR_Q-PAS1_ dimer (based on the PpsR_N-Q-PAS1_ structure) and the corresponding VicK S-helix PAS dimer can be superimposed with an average root-mean-square deviation (r.m.s.d.) of 4.2 Å for 2 × 131 C^α^ atoms, reflecting the similarities in dimeric arrangements (Fig. 5 ▶ *b*). Furthermore, a comparable S-helix PAS organization can also be observed in a truncated structure of the HTR-like protein from *Halo­arcula marismortui* (Fig. 5 ▶ *c*), which resembles the PpsR_Q-PAS1_ structure. A search of the Pfam database (Punta *et al.*, 2012 ▶) revealed that the S-helix also connects two PAS domains in the HTR-like protein, in analogy to the Q-linker of PpsR. However, since additional PAS and effector domains are present in this protein, the quaternary structure of the full-length protein is difficult to predict.

### 3.3. Oligomerization states of PpsR_full_ and the truncated variants   

**Figure 6:**
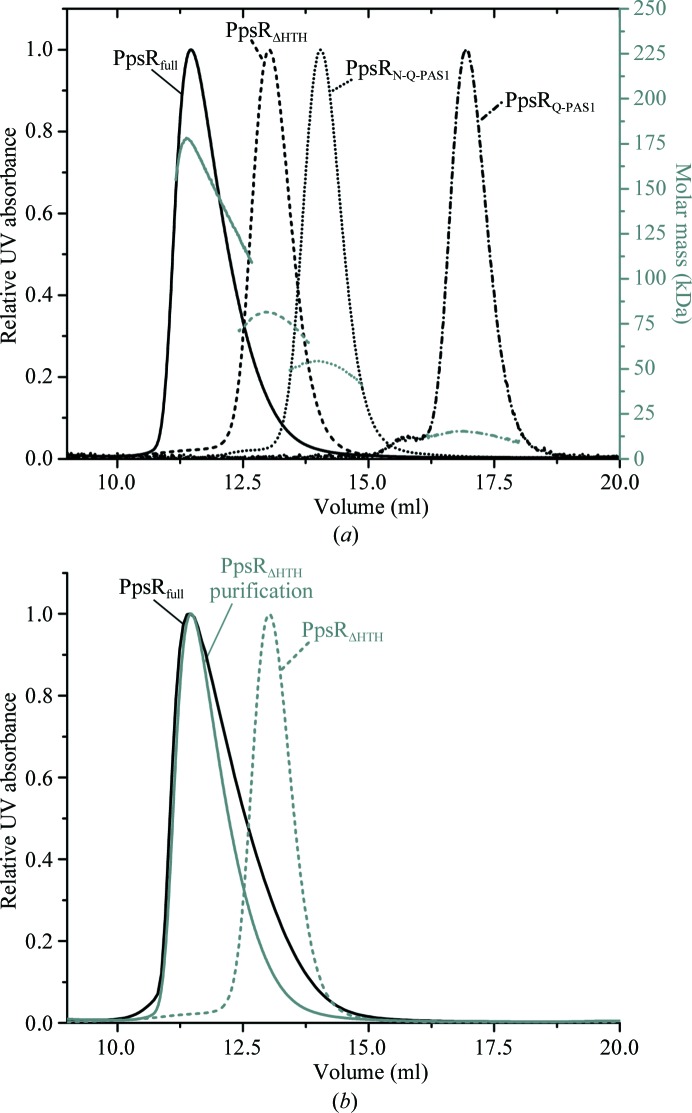
(*a*) Multi-angle light-scattering detection of PpsR_full_ (straight line), PpsR_ΔHTH_ (dashed line), PpsR_N-Q-PAS1_ (dotted line) and PpsR_Q-PAS1_ (dashed/dotted line) fractionated by size-exclusion chromatography (individual traces are normalized according to the absorbance at 280 nm). The MALS-derived calculated molar-mass signals (grey) indicated on the right *y* axis yield average values of 150, 77, 52 and 14.3 kDa, respectively. (*b*) Absorbance traces of PpsR_full_ (black line) and PpsR_ΔHTH_ (grey dashed line) at 280 nm from the multi-angle light-scattering experiment in comparison to the absorbance trace of PpsR_ΔHTH_ (grey solid line) during preparative purification. The PpsR_ΔHTH_ concentration during preparative purification was about 30-fold higher compared with the multi-angle light-scattering experiment.

To better understand the importance of individual PAS domains in oligomerization of PpsR, we determined the molar mass of the crystallized constructs using size-exclusion chromatography coupled to a MALS detector and compared them with PpsR_full_, which has been shown to exist in a dynamic equilibrium of dimers and tetramers in solution (Winkler *et al.*, 2013 ▶). Molar masses of 77 and 52 kDa, respectively, were determined for PpsR_ΔHTH_ and PpsR_N-Q-PAS1_ (Fig. 6 ▶ *a*). In both cases, this corresponds to approximately 1.8 times the molar mass of a monomer (42 kDa for PpsR_ΔHTH_ and 29 kDa for PpsR_N-Q-PAS1_). The eluting peaks did not show pronounced tailing and no continuous decrease in the molar mass signal was detected as observed for PpsR_full_ (Winkler *et al.*, 2013 ▶; Fig. 6 ▶ *a*). Together, these findings indicate that both PpsR variants exist predominantly as dimers under the conditions of this experiment. However, the retention time of PpsR_ΔHTH_ was significantly reduced upon size-exclusion chromatography during protein purification at higher protein concentrations (Fig. 6 ▶ *b*) and the elution profile showed peak tailing as observed for PpsR_full_ (Winkler *et al.*, 2013 ▶). This finding suggests that at higher concentrations the PpsR_ΔHTH_ variant forms tetramers similar to PpsR_full_. However, neither tailing nor a reduction in the elution volume of the eluting peak was observed during PpsR_N-Q-PAS1_ purification, suggesting that PpsR_N-Q-PAS1_ is not able to form tetramers even at high concentrations. In contrast to the other studied PpsR variants, PpsR_Q-PAS1_ exists predominantly as a monomer at the concentrations used in this experiment. The average molar mass of 14.3 kDa determined by MALS is in good agreement with the calculated monomer mass of 15.3 kDa.

**Figure 7:**
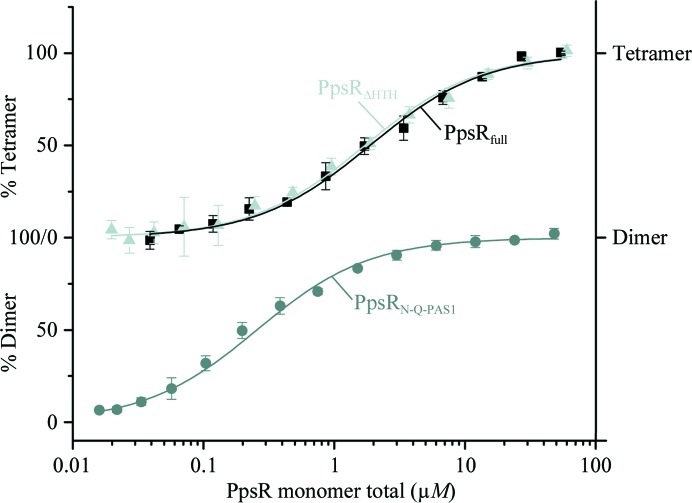
Microscale thermophoresis measurements of the dimer–tetramer equilibria of PpsR_full_ (black line and squares) and PpsR_ΔHTH_ (light grey line and triangles) result in *K*_d_ values of 0.9 ± 0.3 and 0.9 ± 0.2 µ*M*, respectively, based on the total dimer concentration. Measurements of the PpsR_N-Q-PAS1_ monomer–dimer equilibrium (dark grey line and filled circles) result in a *K*_d_ value of 0.25 ± 0.05 µ*M*, based on the total monomer concentration. Error bars represent the standard deviation of three individual experiments.

To analyse and quantify the dimer–tetramer equilibrium of PpsR_ΔHTH_, we performed microscale thermophoresis measurements (Fig. 7 ▶). A *K*_d_ value of 0.9 ± 0.2 µ*M* was determined for 2 (PpsR_ΔHTH_)_2_ ⇄ (PpsR_ΔHTH_)_4_ based on the total dimer concentration. This value is in good agreement with the previously reported *K*_d_ value of 0.9 ± 0.3 µ*M* determined for PpsR_full_ (Winkler *et al.*, 2013 ▶). Although the *K*_d_ values for the dimer–tetramer equilibrium of PpsR_full_ and PpsR_ΔHTH_ are comparable, MALS measurements at comparable concentrations showed such an equilibrium only for PpsR_full_ and not for PpsR_ΔHTH_. This suggests that the HTH domain of PpsR influences the kinetic stability of the tetramer. In addition to the measurements for PpsR_ΔHTH_, we also performed MST measurements for the PpsR_N-Q-PAS1_ and PpsR_Q-PAS1_ variants. In agreement with the observation of exclusively dimeric PpsR_N-Q-PAS1_ in our MALS experiments, we quantified the 2 PpsR_N-Q-PAS1_ ⇄ (PpsR_N-Q-PAS1_)_2_ transition with a *K*_d_ value of 0.25 ± 0.05 µ*M*. In contrast, for PpsR_Q-PAS1_ we could not detect dimerization over the concentration range used in the MST experiments. Nevertheless, the concentrations used, for example, for crystallization also supported the formation of a biologically relevant PpsR_Q-PAS1_ dimer.

## 4. Discussion   

We provide a detailed structural characterization of PpsR from *R. sphaeroides*, which reveals the importance of individual PAS domains and their α-helical extensions for PpsR oligomerization. Whereas the implications of the PpsR_ΔHTH_ structure in the context of the AppA–PpsR_2_ interaction and the regulation of gene expression in *R. sphaeroides* have recently been described (Winkler *et al.*, 2013 ▶); here, we discuss the structural aspects of PpsR, which enable a better understanding of the architecture and functionality of multi-PAS proteins in general. The PpsR_ΔHTH_ structure is the first protein structure with three classical PAS domains present on the same polypeptide chain and reveals an intertwined tetrameric arrangement formed by two head-to-tail dimers. Each dimer is formed by two head-to-head aligned PpsR protomers featuring extensive contacts between all domains. The highly conserved Q-linker connects the N-domain and the PAS1 domain and forms a long coiled-coil-like structure, which is involved in formation of the PpsR dimer as well as the tetramer. As proposed previously, the N-domain also significantly contributes to PpsR oligomer formation and/or integrity (Yamazaki *et al.*, 2008 ▶), most likely by stabilizing the extended Q-linker helix as shown by the disordered N-terminal Q-linker part of the PpsR_Q-PAS1_ structure. PpsR constructs lacking the N-domain are unable to bind to DNA *in vivo* (Gomelsky *et al.*, 2000 ▶) and *in vitro* (Yamazaki *et al.*, 2008 ▶), which highlights the central importance of PpsR oligomerization. In addition to its role in PpsR dimer and tetramer formation, the Q-linker provides an additional oligomerization interface that allows PpsR octamer formation (Fig. 3 ▶ *e*). Such an octameric assembly has been reported to play a role in the binding of PpsR to its palindromic DNA target sequence (Jaubert *et al.*, 2004 ▶; Winkler *et al.*, 2013 ▶). Taken together, this highlights the involvement of the α-helical PAS extensions in the oligomerization and functionality of multi-PAS proteins.

This is also supported by MALS and MST experiments of PpsR_full_ and PpsR_ΔHTH_, which clearly show PpsR tetramer formation in solution. However, the crystal structure does not allow differentiation between tetramers formed by protomers *AB* and *CD* or by two *AB* pairs (*A*_2_ *B*_2_) which are related by a crystallographic twofold axis, since the individual BSAs of the two tetramer assemblies are in comparable ranges. Nevertheless, the *ABCD* tetramer is most likely, since the truncated PpsR_N-Q-PAS1_ variant, which provides the entire interface for an *A*_2_ *B*_2_ tetramer, is not able to form tetramers. In contrast, PpsR_full_ and PpsR_ΔHTH_ show the characteristic dimer–tetramer equilibrium, supporting a role of PAS2 in tetramer formation. Further support for the functional relevance of the *ABCD* tetramer is provided by interactions between the highly conserved helix α3_2_ and the Q-linker (Fig. 2 ▶). It has been demonstrated that the Q-linker is not only involved in PpsR oligomerization but also serves as a binding site for the regulatory protein AppA (Winkler *et al.*, 2013 ▶) that enables the blue-light- and oxygen-dependent transcriptional control of PpsR-regulated genes (Gomelsky & Kaplan, 1998 ▶; Masuda & Bauer, 2002 ▶; Braatsch *et al.*, 2002 ▶; Jäger *et al.*, 2007 ▶; Han *et al.*, 2007 ▶). AppA interacts *via* its four-helix bundle with the PAS2 binding region of the Q-linker to form an AppA–PpsR_2_ complex, thereby preventing PpsR tetramer formation. Hydrogen–deuterium exchange experiments comparing this dimeric state of AppA-complexed PpsR and PpsR predominantly in its tetrameric form further support the biological relevance of the crystallographic tetramer observed in the PpsR_ΔHTH_ structure (Winkler *et al.*, 2013 ▶).

However, the DNA binding of PpsR and its homologues is not exclusively regulated by protein–protein interactions. A variety of regulatory mechanisms involving the partially conserved cysteine (Cys424; Fig. 2 ▶) located close to the HTH motif have been proposed (Masuda *et al.*, 2002 ▶; Masuda & Bauer, 2002 ▶; Jaubert *et al.*, 2004 ▶; Yin *et al.*, 2012 ▶; Cheng *et al.*, 2012 ▶). Depending on the species, this cysteine has been reported to be involved in intermolecular (Jaubert *et al.*, 2004 ▶) or intramolecular (Masuda & Bauer, 2002 ▶; Masuda *et al.*, 2002 ▶) disulfide-bond formation, cofactor binding (Yin *et al.*, 2012 ▶) or as the target for oxidative modifications (Cheng *et al.*, 2012 ▶). Early studies on PpsR from *R. sphaeroides* and the PpsR homologue CrtJ from *R. capsulatus* suggested that redox-dependent DNA binding can be regulated *via* the formation of an intramolecular disulfide bond between the two cysteines located in the HTH and the PAS1 domain (Masuda & Bauer, 2002 ▶; Masuda *et al.*, 2002 ▶; Cho *et al.*, 2004 ▶). However, considering the location of Cys251 within the PAS1 domain, the formation of an intramolecular disulfide bond involving Cys251 appears to be unlikely. Disulfide-bond formation would require significant structural changes of PAS1 and would most probably result in disruption of the PAS fold. Interestingly, Cys251 points into a small cavity inside the PAS1 domain where cofactors can be covalently or noncovalently bound in a variety of PAS proteins.

Like PpsR, many PAS proteins contain multiple PAS domains that either bind cofactors and sense environmental stimuli or exclusively serve as protein-interaction or signal transmission modules. Therefore, multi-PAS domain-mediated protein–protein interaction and oligomerization, as observed for PpsR, appears to be a common feature of many PAS-containing proteins. The PpsR_ΔHTH_ structure highlights this function and shows that not only the PAS core regions, but also the N- and C-terminal α-helical extensions, can significantly contribute to protein oligomer formation. Structural studies of PAS proteins such as *Rm*FixL (Miyatake *et al.*, 2000 ▶), *Ec*Dos (Kurokawa *et al.*, 2004 ▶), *Av*NifL (PAS1; Key *et al.*, 2007 ▶), *Ec*TyrR (Verger *et al.*, 2007 ▶), *Np*STHK (Ma *et al.*, 2008 ▶), *Mt*Rv1364c (Jaiswal *et al.*, 2010 ▶) and *Sm*VicK (Wang *et al.*, 2013 ▶) revealed that structures containing N-caps frequently form homodimers that exhibit a parallel arrangement similar to the three PAS domains of PpsR. In contrast, structures lacking N-terminal helices [*Bj*FixLH (Ayers & Moffat, 2008 ▶), *Bs*YtvA-LOV (Möglich & Moffat, 2007 ▶) and *Cr*Phot-LOV1 (Fedorov *et al.*, 2003 ▶)] exhibit diverse quaternary assemblies (Ayers & Moffat, 2008 ▶). The important role of N-caps in PAS dimer formation is also supported by the crystal structure of the heterodimeric mouse CLOCK–BMAL1 transcriptional activator complex (Huang *et al.*, 2012 ▶). The PAS-A domains of CLOCK and BMAL1, which both possess N-caps, heterodimerize in an arrangement similar to the PAS domains of PpsR. In contrast, their PAS-B domains as well as the PAS-B domains of HIF-2α and ARNT (Scheuermann *et al.*, 2009 ▶), which lack N-caps, dimerize *via* different mechanisms. Additionally, studies of the PAS domain of *Np*STHK (termed H-NOXA) show that the deletion of seven N-cap residues disrupts the parallel dimer arrangement and results in a flipped, most likely nonphysiological, dimer (Ma *et al.*, 2008 ▶). Similarly, structural and functional studies on KinA have also shown the importance of the N-terminal PAS_B_ extension for histidine kinase activity (Winnen *et al.*, 2013 ▶). Even mutations within the N-cap can influence the quaternary structure of PAS proteins and thus have a strong impact on protein function. It has recently been shown for the CLOCK–BMAL1 PAS-A system that mutation of two residues at the interface of the N-cap dimer interferes with heterodimerization and significantly reduces transactivation activity (Huang *et al.*, 2012 ▶). Also, single substitutions within the N-cap of the histidine kinases *Sp*MicB (Echenique & Trombe, 2001 ▶), *Ec*DcuS (PAS_C_; Monzel *et al.*, 2013 ▶) and the histidine kinase-like protein *Av*NifL (PAS1; Little *et al.*, 2012 ▶) can affect protein function, most likely owing to impaired dimerization of the proteins. Additionally, N-cap mutations can affect cofactor binding and thus PAS-mediated signalling, as shown for the aerotaxis receptor *Ec*Aer (Watts *et al.*, 2006 ▶). The important function of N-caps in signal transduction and protein oligomerization is further supported by studies on the LOV protein VIVID from *Neurospora crassa* (VVD). Blue-light illumination of VVD results in a series of structural changes that eventually affect the N-cap conformation and thus induce VVD dimerization (Zoltowski *et al.*, 2007 ▶; Zoltowski & Crane, 2008 ▶; Vaidya *et al.*, 2011 ▶). However, not all PAS domains that possess N-caps dimerize in an assembly as observed for PpsR and the proteins mentioned above. The sensory histidine kinases DctB of *Sinorhizobium meliloti* (Zhou *et al.*, 2008 ▶) and DcuS (PAS_P_) of *E. coli* (Cheung & Hendrickson, 2008 ▶), for example, mainly dimerize *via* a long helix preceding the classical N-cap, whereas the core regions of their PAS domains do not contribute to dimer formation. However, in the cases of DctB and DcuS (PAS_P_) the PAS N-­caps also help to stabilize the homodimer in its ligand-free and ligand-bound state, respectively.

Helices flanking the PAS core are observed at the N-­terminus but also at the C-terminus of PAS domains. Protein-sequence analysis suggests that these α-helical extensions are frequently much longer than classical N-caps and often form coiled coils (Anantharaman *et al.*, 2006 ▶; Möglich *et al.*, 2009*b* ▶) similar to the Q-linker of PpsR. These long helices, termed S-helices (Anantharaman *et al.*, 2006 ▶), occur in a wide range of multi-domain signalling proteins and frequently link PAS to effector domains, PAS and sensory modules as well as tandem PAS domains. It has been proposed that the coiled-coil S-helices play an important role in signal propagation from sensory domains to effector domains or other sensory domains (Anantharaman *et al.*, 2006 ▶; Möglich *et al.*, 2009*a* ▶; Hao *et al.*, 2011 ▶; Little *et al.*, 2012 ▶). Additionally, they can be involved in the control of protein oligomerization by serving as binding sites for regulatory proteins, as shown for the binding of AppA to the Q-linker of PpsR (Winkler *et al.*, 2013 ▶). Upon binding, AppA induces an overall asymmetry in the AppA–PpsR_2_ complex that results in structural changes of the N-terminal part of one Q-linker, whereas the conformation of the C-terminal part, which corresponds to one N-cap of the PAS1 dimer, and the N-­domain dimer remain unaffected and resemble that observed in other PpsR structures (Fig. 5 ▶ *a*). This suggests that the PAS domain dimers together with their N-caps function as rigid modules. The flexibility of a defined hinge region within the Q-linkers, however, supports the finding that the long Q-­linker helices observed in the PpsR_ΔHTH_ structure result from a direct fusion of the C-­terminal extensions of the N-­domains with the N-cap of the PAS1 domains. Similar to the Q-linker of PpsR, *Sm*VicK also possesses a long S-helix that originates from a fusion of a HAMP helix (as commonly found in **h**istidine kinases, adenylyl cyclases, methyl-accepting chemotaxis proteins and phosphatases) with the N-cap of a PAS domain (Wang *et al.*, 2013 ▶). The S-helix–PAS dimer arrangement observed for *Sm*VicK is nearly identical to that observed for the PpsR_ΔHTH_ Q-PAS1 dimer (Fig. 5 ▶ *b*). Additionally, a similar S-helix PAS dimer arrangement is also observed for the HTR-like protein from *H. marismortui* (Fig. 5 ▶ *c*; PDB entry 3bwl; Midwest Center for Structural Genomics, unpublished work). Although the N-terminal region of the S-helix and the adjacent PAS domain are not part of the HTR-like protein crystal structure, it is likely that the dimer arrangement of tandem PAS domains featuring characteristic N-caps is similar to that observed for PpsR_N-Q-PAS1_. Therefore, the PAS–S-­helix–PAS dimer organ­ization observed for PspR might not only be a conserved dimerization motif in a variety of multi-PAS containing signalling proteins, but might also be a general architecture for the connection of PAS domains to a variety of effector and sensory domains such as REC, GAF, HAMP, MEDS or PocR domains. For the engineered light-regulated sensor histidine kinase YF1, where an S-helix connects a LOV light-sensor to a dimerization/histidine phosphotransfer domain, it has been proposed that the sensed signal is transmitted along the coiled-coil S-helices in the form of torque or helical rotation (Möglich *et al.*, 2009*a* ▶; Diensthuber *et al.*, 2013 ▶). Additionally, the same mechanism has also been proposed for the sensory protein *Av*NifL (Little *et al.*, 2012 ▶), which exhibits a PAS–S-­helix–PAS dimer organization as observed for PpsR. Also, upon AppA–PpsR_2_ complex formation a pronounced rotation of the PAS1 domain dimer along the Q-linker axis was observed (Winkler *et al.*, 2013 ▶). Therefore, it can be suggested that in many PAS-containing proteins signals are transmitted along S-helices *via* such a mechanism.

However, C-terminal PAS extensions do not always extend from the PAS core and/or form coiled coils. They can also interact with the surface of the PAS β-sheet, as observed for the Jα helix in the *Avena sativa* phototropin 1 PAS B (LOV2) domain (Halavaty & Moffat, 2007 ▶). Also in the case of LOV2, the C-terminal α-helix is proposed to play an important role in signal transduction by transmitting light-induced structural changes from the PAS core to the kinase domain (Harper *et al.*, 2003 ▶, 2004 ▶; Halavaty & Moffat, 2007 ▶). Additionally, C-­terminal PAS extensions can interact with the helical part of the PAS core, as observed for the αE helix of the *Drosophila* protein PERIOD (Yildiz *et al.*, 2005 ▶; King *et al.*, 2011 ▶) and its three mouse homologues mPER1 (Kucera *et al.*, 2012 ▶), mPER2 (Hennig *et al.*, 2009 ▶) and mPER3 (Kucera *et al.*, 2012 ▶). Taken together, although the structural details of the N-­terminal and C-terminal PAS extensions can vary between different proteins, they play an important role in PAS-mediated protein oligomerization and signal transduction.

To better understand signal transduction from PAS to effector domains, as well as the interplay between multiple PAS domains and their N-terminal and C-terminal α-helical extensions in protein oligomerization, structures of full-length proteins are required. However, owing to the inherent flexibility of multi-domain PAS signalling proteins, their crystallization and structure determination is challenging. For the first time, the determined PpsR structures reveal how three PAS domains within a single peptide chain, together with their N-terminal and C-terminal α-helical extensions, enable the formation of multiple oligomeric states (dimer, tetramer, octamer). Additionally, the PAS–S-helix–PAS dimer organization observed for PpsR appears to be a common architecture for the connection of PAS domains to a variety of effector and sensory domains in multi-domain signalling proteins. The signals sensed by such PAS domains are transmitted *via* S-­helices to effector or other sensory domains, thus enabling PAS domains to regulate the activity of a variety of different effector domains without directly interacting with them. Considering that the oligomerization states of PAS domain-containing proteins can be regulated by their cellular abundance or regulatory proteins, changes in quaternary structure as well as the modulation of the homo- or hetero-oligomerization affinity by cofactor binding appear to be general aspects of PAS domains and to be an essential feature for efficient signal processing and/or transduction.

## Supplementary Material

PDB reference: PpsR_Q-PAS1_, 4l9e

PDB reference: PpsR_Q-PAS1_ SeMet, 4l9f

PDB reference: PpsR_N-Q-PAS1_, 4l9g

Supplementary Figures S1-S3. DOI: 10.1107/S1399004713033634/cb5045sup5.pdf

## Figures and Tables

**Table 1:** Data-collection and refinement statistics The statistics for PpsR_HTH_ and PpsR_HTH_ Se-SAD have recently been published (Winkler *et al.*, 2013 ▶). Values in parentheses are for the highest resolution shell.

	PpsR_Q-PAS1_ Se-SAD†	PpsR_Q-PAS1_	PpsR_N-Q-PAS1_
Data collection			
Space group	*P*6_1_22	*P*6_1_22	*P*2_1_2_1_2
Unit-cell parameters ()			
*a* ()	50.6	50.4	50.4
*b* ()	50.6	50.4	107.9
*c* ()	163.5	163.3	92.1
= ()	90.0	90.0	90.0
()	120.0	120.0	90.0
Wavelength ()	0.9794	0.9765	0.9785
Resolution range ()	502.5 (2.602.50)	501.65 (1.701.65)	502.2 (2.302.20)
Unique reflections	8108 (887)‡	15633 (1299)	26156 (3183)
Completeness (%)	100 (99.8)	99.9 (100)	99.8 (99.7)
Multiplicity	40.8 (38.6)	12.6 (12.7)	4.3 (4.2)
*R*_merge_ (%)	14.8 (77.0)	4.9 (55.8)	10.4 (63.1)
*I*/(*I*)	26.87 (6.43)	21.06 (4.42)	10.37 (2.38)
Wilson *B* (^2^)	36.0	33.2	30.1
Refinement			
Resolution range ()	42.32.5	43.61.65	46.62.2
No. of reflections	4783	15631	26156
*R*_work_/*R*_free_ § (%)	0.1930/0.2373	0.1967/0.2190	0.1767/0.2332
No. of atoms			
Protein	861	894	3862
Ligand/ion			32
Water	22	85	147
Total	883	979	4041
R.m.s. deviations			
Bond lengths ()	0.009	0.006	0.010
Bond angles ()	1.192	0.966	1.209
*B* factors			
Protein	41.4	41.7	44.0
Ligand/ion			65.5
Water	40.5	49.7	43.6
Average	41.3	42.4	44.2
Ramachandran statistics (%)			
Favoured	98.1	99.1	98.2
Allowed	1.9	0.9	1.8
Outliers	0	0	0

† Statistics reported to the cutoff used for anomalous data processing.

‡ Friedel pairs were treated as separate reflections in this case.

§ *R*_free_ values were calculated using a randomly assigned 5% of the reflections, which were omitted during refinement.

## Footnotes

1 Supporting information has been deposited in the IUCr electronic archive (Reference: CB5045).
