# Supplementary material for: Multi-PAS domain-mediated protein oligomerization of PpsR from Rhodobacter sphaeroides
Source: Acta Crystallogr D Biol Crystallogr. 2014 Feb 27;70(Pt 3):863–76. doi: 10.1107/S1399004713033634 (PMC3949515; doi:10.1107/S1399004713033634)
Supplement: Supplementary file 1 [file d-70-00863-sup5.pdf]

<u>Supplementary Information</u>

for

Multi-PAS domain mediated protein oligomerisation of PpsR from *Rhodobacter sphaeroides*

Udo Heintz, Anton Meinhart and Andreas Winkler*

Department of Biomolecular Mechanisms, Max Planck Institute for Medical Research, Heidelberg, Germany.

Correspondence should be addressed to A.W. (Andreas.Winkler@mpimf-heidelberg.mpg.de)

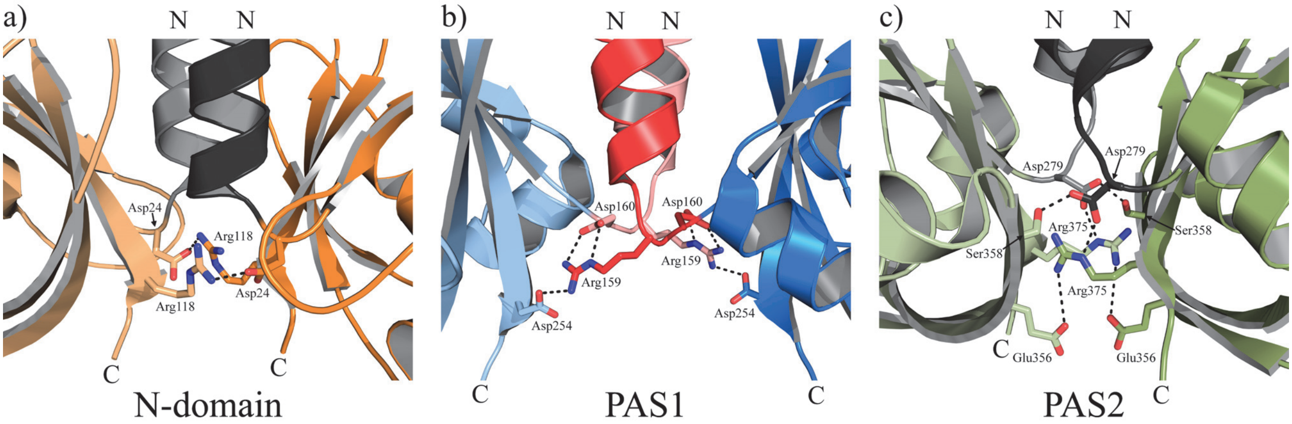

Supplementary Figure 1 Close-up view of the three different PpsR PAS domain dimer interfaces (*cf*. Figure 4). Conserved amino acids involved in inter-protomer contacts are shown as stick models. Dashed lines indicate hydrogen bonds or salt-bridges. The PAS core and N-cap of one protomer is coloured according to Figure 3 d protomer C, whereas the second protomer is coloured in equivalent but lighter colours. a) N-domain dimer (residues 6-120). b) PAS1 dimer (residues 147-256). c) PAS2 dimer (residues 264-378). N- and C-termini of each domain are labelled.

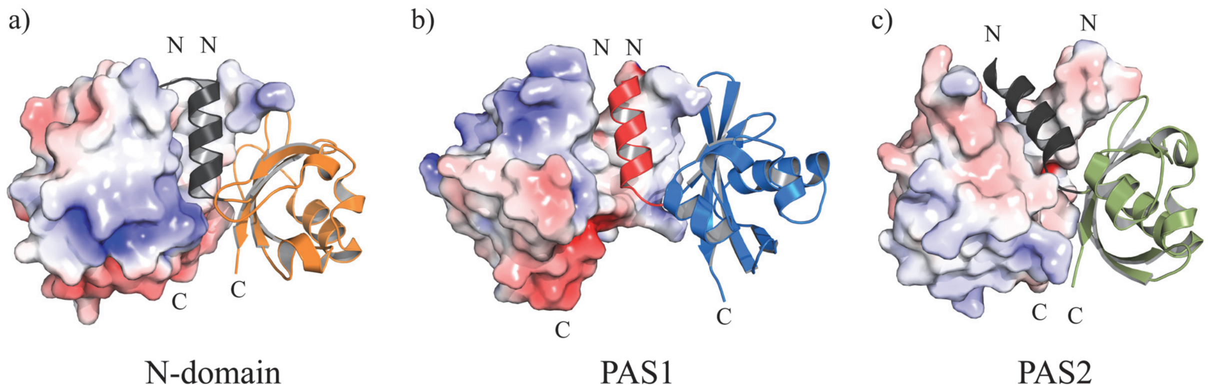

Supplementary Figure 2 Structure of the three different PpsR PAS domain dimers including their N-caps (*cf*. Figure 4). One protomer of each dimer is shown in surface representation coloured according to the electrostatic surface potential on a scale from -6 kT to 6 kT for regions of negative (red) and positive (blue) electrostatic potentials, respectively, whereas the second protomer is shown in cartoon representation. The PAS cores and N-caps of the cartoon models are coloured according to Figure 3 d protomer C. The N-caps of the different PAS domains significantly contribute to dimer formation by protecting hydrophobic patches on the PAS cores. a) N-domain dimer (residues 6-120). b) PAS1 dimer (residues 147-256). c) PAS2 dimer (residues 264-378). N- and C-termini of each domain are labelled.

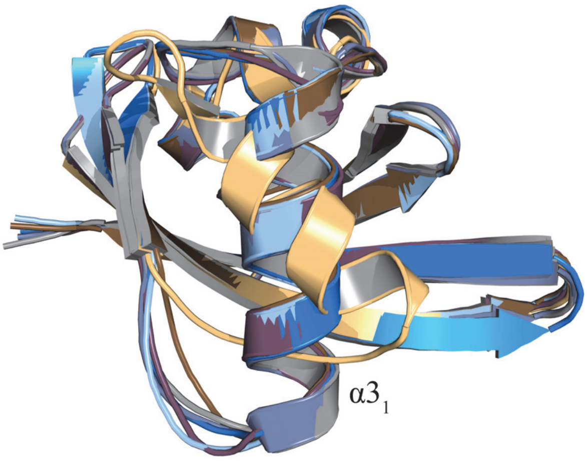

Supplementary Figure 3 Superposition of all modelled PpsR PAS1 domains. The colour coding of the PAS1 domains is according to Figure 3 c, d, e ($PpsR_{\Delta HTH}$ - protomer A, light-orange; B, gray; C, slate; D, dark-purple; $PpsR_{N\text{-}Q\text{-}PAS1}$ - protomer A, blue; B, light-blue) and the PAS1 domain of $PpsR_{Q\text{-}PAS1}$ is coloured in brown. Helix $\alpha 3_1$ of the PAS1 domain of $PpsR_{\Delta HTH}$ protomer A (light-orange) is rotated by about 42° compared to all other PAS1 domains.
